# Magnetic properties, magnetocaloric effect, and critical behaviors in Co_1−*x*_Cr_*x*_Fe_2_O_4_

**DOI:** 10.1039/d2ra02223k

**Published:** 2022-06-13

**Authors:** M. A. Islam, A. K. M. Akther Hossain

**Affiliations:** Department of Physics, Bangladesh University of Engineering and Technology Dhaka-1000 Bangladesh akmhossain@phy.buet.ac.bd

## Abstract

This research work focuses on the magnetic properties, nature of the magnetic phase transition, magnetocaloric effect, and critical scaling of magnetization of various Co_1−*x*_Cr_*x*_Fe_2_O_4_ (*x* = 0, 0.125, 0.25, 0.375, and 0.5). The tunability of the magnetic moment, exchange interactions, magnetocrystalline anisotropy constant, and microwave frequency using Cr^3+^ content has been found. The nature of the magnetic phase transitions for all the Cr^3+^ concentrations exhibits as second order which has been confirmed from the analysis of critical scaling, universal curve scaling, and scaling analysis of the magnetocaloric effect. The critical exponent analysis for all samples was performed from the modified Arrott-, and Kouvel–Fisher-plots. These critical analyses suggest that *x* = 0.125, 0.250, and 0.375 samples show reliable results in the magnetocaloric effect with relative cooling power (RCP) values in the range of 128–145 J kg^−1^. On the other hand, *x* = 0.00, and 0.500 samples exhibit inconsistent RCP values. The universal curve scaling also confirms the reliability of the magnetocaloric effect of the investigated samples.

## Abstract 2

Magnetic entropy change as a function of temperature for various Co_1−*x*_Cr_*x*_Fe_2_O_4_ at an applied magnetic field of 5 T.

## Graphical abstract

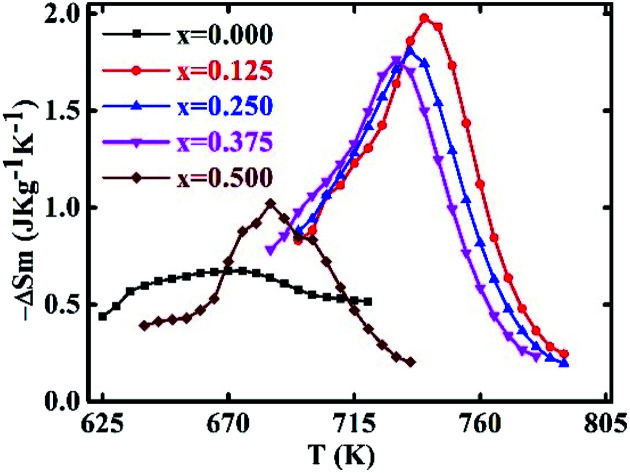

## 1 Introduction

Over the past few decades, research on iron oxide compounds like spinel ferrite, hexaferrite, and garnet has become a topic of discussion among scientists, due to their attractive practical applications in magneto-sensing devices, and biotechnology.^1–7^ These key topics attracted scientists because these types of compounds exhibit unique magnetic and magnetocaloric effects (MCE).^1–3,8–10^ Among all the ferrites, CoFe_2_O_4_ (COF) has been focused on in recent years by academia, the medical sector, and industry due to its remarkable magnetic and MCE properties.^11–19^ The structural, magnetic, and MCE properties of COF can be tuned by doping/substituting divalent or trivalent cations.^11,18–24^ The tunability of structural parameters due to Cr^3+^ substitution in stoichiometric and non-stoichiometric COF is reported in our earlier literature.^25^ It was observed that there are structural defects due to Cr^3+^ substitution. In recent years, a large number of articles have been found on the study of magnetic field (*H*) and temperature (*T*) dependent magnetization (*M*) of various ferrites.^1,4,26,27^ Massoudi *et al.* have observed the non-collinear model on Ni–Zn–Al ferrite by comparing the theoretical and experimental magnetic moment calculated from the cation distribution and M–H hysteresis curve, respectively.^1^ The paramagnetic moment followed by the Curie–Weiss law and magnetic phase transition temperature has been studied from the field cooled (FC) and zero fields cooled (ZFC) magnetization.^23,28^ Spinel ferrites also attracted scientists across the world due to their interesting MCE properties.^4,29–32^ The MCE is an intrinsic thermodynamic property of magnetic materials that causes a change in the temperature of the substance under the action of a magnetic field.^33^ In various literature, the MCE of such materials has been studied from the isothermal M–H over a wide temperature range near the magnetic phase transition.^4,34^ The MCE values have been calculated from the change of magnetic entropy from the isothermal M–H curve based on Maxwell's thermodynamic relation.^34,35^ The nature of magnetic phase transition has also been reported in various literature extracted from the isothermal M–H curves using the Arrott plot,^36^ and the Arrott–Noakes model.^37^ Law *et al.* have studied the nature of magnetic phase transition by calculating a critical exponent *n* from the change of entropy as a function of temperature.^38^ Various reports have been conducted on the analysis of critical exponents to confirm the universal class of materials.^32,34^ Franco *et al.* have first reported the phenomenological universal scaling curve taking the normalized entropy change as a function of rescaled temperature.^39^

In this research, the effect of Cr^3+^ substitution on magnetic and MCE properties of various Co_1−*x*_Cr_*x*_Fe_2_O_4_ (*x* = 0, 0.125, 0.25, 0.375, and 0.5) have been studied. A detailed investigation of magnetic properties has been carried out by analyzing the M–H hysteresis and FC-ZFC magnetization behaviors. The MCE properties of Cr^3+^ substituted cobalt ferrite have been investigated by analyzing the M–H isotherms. The nature of magnetic phase transition has also been examined by analyzing the Arrott plot. The nature of the universal class of these materials has been analyzed by calculating the critical exponent followed by the modified Arrott plot (MAP), the Kouvel–Fisher method, and critical isotherm analysis. Finally, the reliability of the MCE properties and universal class have been studied in detail using as usual methods.

## 2 Experimental

The nominal chemical compositions Co_1−*x*_Cr_*x*_Fe_2_O_4_ (*x* = 0, 0.125, 0.25, 0.375, and 0.5) have been synthesized by the standard solid–state reaction technique. The stoichiometric amount of Co_2_O_3_ (98.0%), Cr_2_O_3_ (99.9%), and Fe_2_O_3_ (96.0%) have been mixed in a mortar with a pestle. After completing the mixing process for 2 hours for each composition, the mixtures were crushed using a planetary ball mill (MSK-SFM-1) for 12 h. To complete the solid–state reaction the milled powder has been calcined at 800 °C for 6 h. Then the calcined powder of each composition has been pressed in the form of a pellet using a uniaxial pressure of 16 000 psi and then sintered at 1200 °C for 6 h. Then a part of sintered pellets was re-crushed into fine powder for performing X-ray diffraction (XRD) to confirm the formation of spinel-type ferrite. The results of phase formation have been reported elsewhere.^25^ After confirming the formation of spinel-type ferrites, these compositions are subjected to further investigation of their magnetic properties. The FC and ZFC magnetization were performed for the measurement of the phase transition temperature. The M–H hysteresis loop measurements were performed at room temperature for saturation magnetization and other relevant parameters, The M–H isotherms at a various temperatures above and below the magnetic phase transition for each composition have been conducted by using Quantum Design MPMS3 SQUID magnetometer. Then the MCE properties and critical scaling have been analyzed for each composition using standard method described in Section 3.4.

## 3 Results and discussion

### 3.1 Structural analysis

The all samples of Co_1−*x*_Cr_*x*_Fe_2_O_4_ exhibit single-phase cubic spinel structure with a space group of *Fd*3̄*m*. The details of crystal structure, with cation distribution, have been explored, and results are already published in ref. 25.

### 3.2 Magnetic properties

**Fig. 1:**
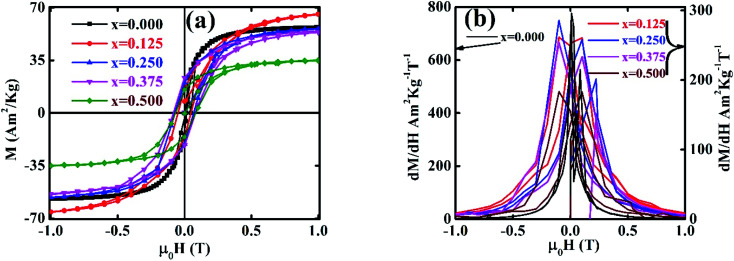
(a) The room temperature M–H hysteresis loops for various Co_1−*x*_Cr_*x*_Fe_2_O_4_. (b) The d*M*/d*H versus H* plots for various Co_1−*x*_Cr_*x*_Fe_2_O_4_.

The saturation magnetization (*M*_s_), remanent magnetization (*M*_r_), and coercivity (*H*_c_) are the most important parameters for a material to know its magnetic behavior. In general magnetization *vs.* applied magnetic field (M–) hysteresis loop provide a reliable information about *M*_s_, *M*_r_, and *H*_c_. The M–H hysteresis loops for all samples have been illustrated in Fig. 1(a). From M–H hysteresis loops the values of *M*_s_, *M*_r_, and *H*_c_ are extracted and listed in Table 1. From the Table 1, it evident that there is a decreasing trend of *M*_s_ with increasing Cr^3+^ content. However, *H*_c_ and *M*_r_ show the increasing trend up to *x* = 0.375 then it decreases for further increase of *x*. The decreasing trend of *M*_s_ may be due to the abnormal grain growth and pore blockage. The increasing trend of *H*_c_ is perhaps due to the decrease in crystallite size (*D*) as calculated from the XRD data.^25^ For *x* = 0.500, the *H*_c_ value does not shows the corresponding behavior as crystallite size which may be due to the excess ion as explained in the literature. To know the inter-grain exchange mechanism, the calculation of remanence ratio *R* (= *M*_r_/*M*_s_) is most important. The calculated *R* values (Table 1) show less than 0.5 which indicates the existence of magnetic dipole interaction with random orientation.^40^ According to Stoner–Wohlfarth theory, the anisotropy constant (*K*) value is related to the coercivity has been calculated using the following expression:^41^1
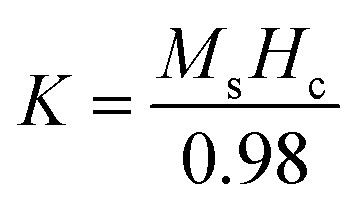


**Table 1:** Magnetic parameters obtained from M–H hysteresis loops, and M–T curve for various Co_1−*x*_Cr_*x*_Fe_2_O_4_

Various parameters	*x*				
	0.000	0.125	0.250	0.375	0.500
*θ*_cw_ (K)	636	720	710	707	810
*C*	2.46	1.15	0.89	0.84	0.35
*T*_C_ (K)	675	740	735	731	687
*T*_B_ (K)	631	685	680	677	671
*M*_s_ (A m^2^ kg^−1^)	58	70	59	57	37
*M*_r_ (A m^2^ kg^−1^)	01	19	20	23	15
*H*_c_ (T)	0.02	0.05	0.07	0.08	0.07
*K* (J m^−3^)	1.44	3.6	4.2	4.6	2.2
*σ*_w_ (J)	6 × 10^−6^	9 × 10^−6^	10 × 10^−6^	11 × 10^−6^	8 × 10^−6^
*D*_m_ (nm)	78	33	32	31	26
*R*	0.02	0.268	0.273	0.277	0.306
*M*_A_ (*μ*_B_)	5.8	5.7	5.6	5.5	5.4
*M*_B_ (*μ*_B_)	10.01	10.07	10.17	10.25	10.34
*n*_B_ (*μ*_B_)	2.45	2.94	2.45	2.37	1.51
*n*_th_ (*μ*_B_)	4.23	4.37	4.56	4.74	4.95
*μ* ^exp^_eff_ (*μ*_B_)	4.43	3.03	2.66	2.59	1.71
*α*_YK_ (deg.)	35	31	38	40	48
*J* (J k^−1^)	1.55 × 10^−21^	1.7 × 10^−21^	1.69 × 10^−21^	1.68 × 10^−21^	1.58 × 10^−21^
*ω*_m_ (GHz)	12.9	15.6	13.0	12.6	8.1

The calculated *K* values for all the Cr concentrations are tabulated in Table 1. It is observed from the Table 1 that *K* values increase with increasing Cr content up to *x* = 0.375, indicating the increase of domain wall energy. Then it shows the decreasing value which may be due to the excess ions showing negative values of the vacancy parameter as explained in the literature.^25^ The domain wall energy (*σ*_w_) can be calculated using the following expression:^42^2
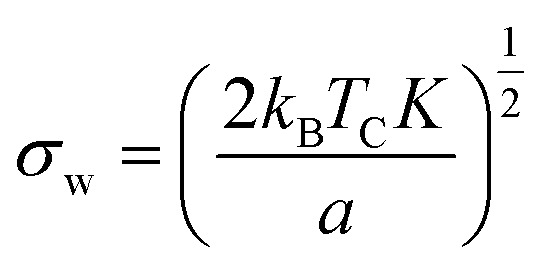
where *k*_B_ is Boltzmann constant, *T*_C_ is Curie temperature and *a* is the lattice constant. The calculated domain wall energy for all samples has been tabulated in Table 1. From Table 1 the values for *σ*_w_ are found to be increasing with the increase of Cr content up to *x* = 0.375 then it shows a decreasing trend.

To know the domain type of materials, the illustration of the d*M*/d*H versus H* plot is most important.^40^ The d*M*/d*H versus H* for all samples have been depicted in Fig. 1(b). Multiple broad peaks near the zero magnetic field observed for all samples indicate multi magnetic domain. To know the agreeable domain nature, determination of critical size by using the following expression is most important:^43^3
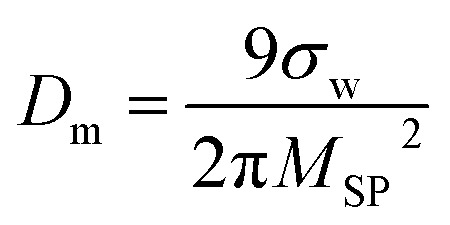
where, *M*_SP_ is spontaneous magnetization. For all samples *D*_m_ (Table 1) shows a lower value than the calculated *D* values from XRD,^25^ which follows the particle spherical model. The *D*_m_ < *D* for all samples reveals that the nanocrystallites have an incipient structure of magnetic domains.^43^

The cation distribution results as presented in our previous article^25^ clearly indicate that both Co^2+^ and Co^3+^ occupy the tetrahedral (A) and octahedral (B) sites, respectively whereas Fe^3+^ occupied both the A- and B-sites for *x* = 0. However, for *x* = 0.125 to 0.500, the Cr^3+^ is found in both the A- and B-sites in place of Co^2+^ and Co^3+^, respectively. The calculated magnetic moment *M*_A_ and *M*_B_ for A- and B-sites are tabulated in Table 1. From Table 1 the values of *M*_A_ are found to be decreasing with an increase of Cr^3+^ which is due to the less magnetic moment of Cr^3+^ (3.87 *μ*_B_) than Co^2+^ (4.87 *μ*_B_). But the values of *M*_B_ are found to be increasing due to an increase of Fe^3+^ with a magnetic moment of 5.92 *μ*_B_. The net theoretical magnetic moment calculated by using *n*_th_ = *M*_B_ − *M*_A_ relation accordingly to Néel's co-linear model is tabulated in Table 1. From Table 1, it is observed that the net magnetic moment show an increasing trend which shows inconsistency with the experimental *M*_s_. To know the reason behind the inconsistency the experimental number of Bohr magneton (*n*_B_) is calculated from the value of *M*_s_ using the following expression:^1^4
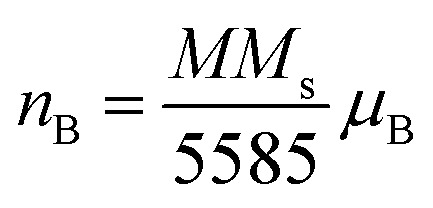
where, *M* is the molecular weight. The calculated values of *n*_B_ are also tabulated in Table 1, where lower values of *n*_B_ compared to that of *n*_th_ are evident which suggests that Néel's collinear model is not agreeable for the synthesized samples. For this reason, Yafet–Kittel (YK) non-collinear model is considered to explain the deviation between *n*_th_ and *n*_B_. According to the YK non-collinear model, the Yafet–Kittel angle (*α*_YK_) can be calculated using the following equation:^1^5
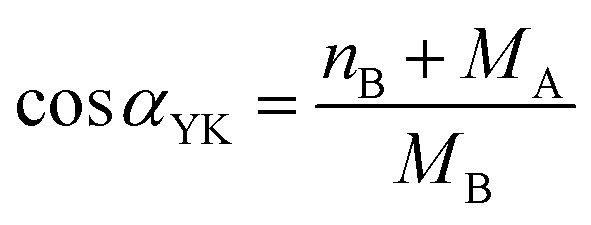


**Fig. 2:**
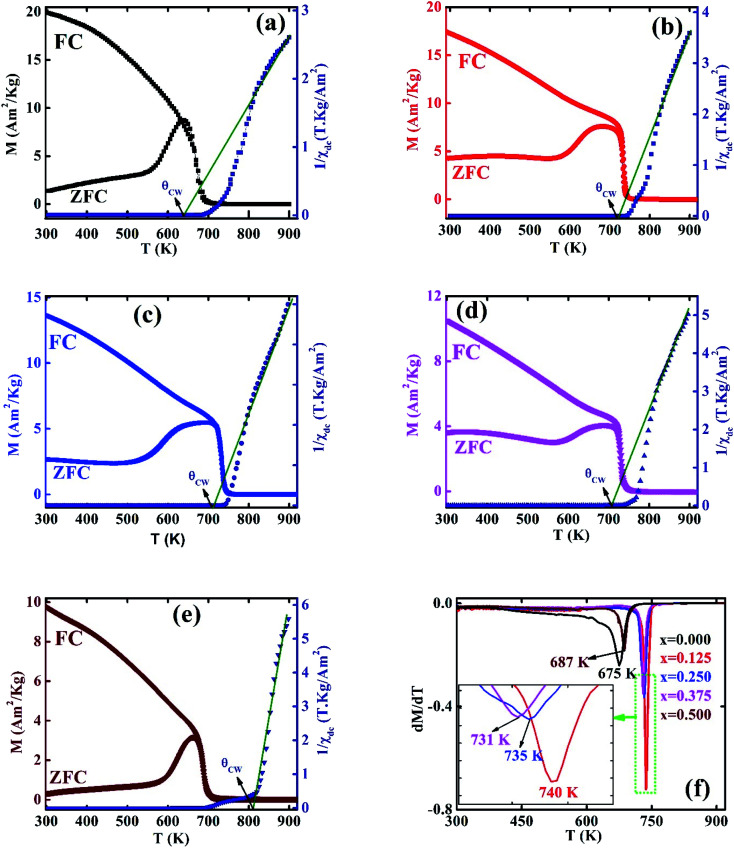
The temperature dependence of FC and ZFC magnetization (left axis) and the inverse susceptibility (right axis) for various Co_1−*x*_Cr_*x*_Fe_2_O_4_ (a) *x* = 0.0, (b) *x* = 0.125, (c) 0.25, (d) 0.375, and (e) *x* = 0.500. (f) The d*M*/d*T vs. T* plots for various Co_1−*x*_Cr_*x*_Fe_2_O_4_.

The *α*_YK_ values for all the samples are found to be in the range of 30 to 50° (Table 1) which confirms the non-collinear spin structure that indicates triangular spin arrangement in the B-sites. The lower values of Cr^3+^ substitution indicate the decreasing trend of *α*_YK_ but at higher values of Cr^3+^ enhance the *α*_YK_. Although decreasing and increasing trends are evident but they do not show zero *α*_YK_. Therefore, the nonzero YK angle suggest that synthesized samples show YK magnetic ordering. The variation of *α*_YK_ with Cr concentration also support the change in Curie temperature (*T*_C_) as evident from Fig. 2.

The FC and ZFC magnetization plots for all samples were recorded in the presence of 10 mT field in the temperature range of 300–900 K as shown in Fig. 2 (a–e left *Y* axis). It is evident that the magnetization (*M*) value in case of ZFC increases up to maximum at a certain temperature for all samples called blocking temperature (*T*_B_) then it shows a decreasing trend with an increase of temperature while the FC magnetization decreases very slowly up to *T*_C_, then a sharp fall is observed for both cases. The values of *T*_B_ of all samples are tabulated in Table 1 where maximum *T*_B_ value is observed for *x* = 0.125. To know the exact *T*_C_ values d*M*/d*T vs. T* graphs are illustrated in Fig. 2(f), where a single peak for all samples confirms the single transition at *T*_C_ without showing any spin frustration. The *T*_C_ values are presented in Table 1. It is observed that *T*_C_ show a maximum for *x* = 0.125. With an increase in Cr content, there is a decrease in *T*_C_ values. The variation of *T*_C_ values and *α*_YK_ angles of these comositions may be explained by the increasing and decreasing trend of calculated exchange interaction (*J*) using the following equation:6
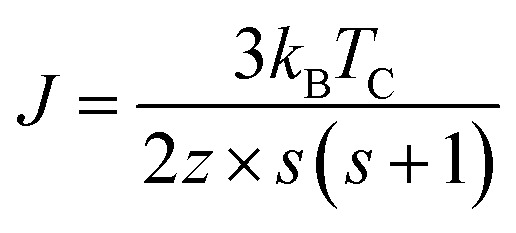
where *z* is the coordination number (= 12) and *s* = ½. The values of *J* are presented in Table 1.

The inverse magnetic susceptibility (*χ*^−1^) as a function of temperature (*T*) is depicted in Fig. 2 (a–e right *Y* axis) for all samples. From Fig. 2(a–e) it is found that the *χ*^−1^ rises sharply when the magnetic state changes from the ferromagnetic to paramagnetic. In the paramagnetic region, susceptibility data follow the Curie–Weiss (CW) expression^34^7
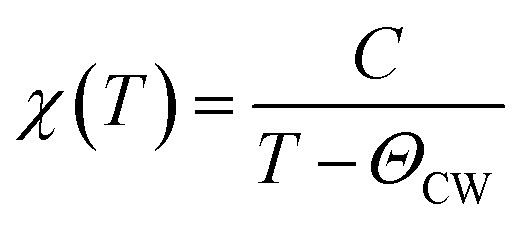
where *C* is the Curie constant which can be obtained from the slope of the linear fit of *χ*^−1^*vs. T* graph (Fig. 2) and *Θ*_CW_ is the CW temperature that also can be obtained from Fig. 2. The calculated values of *C*, and the estimated values of *Θ*_CW_ from the graphs are listed in Table 1. The estimated values *Θ*_CW_ are found to be lower than that of *T*_C_ for the compositions up to *x* = 0.375 which corresponds to the presence of long-range order. However, for *x* = 0.500 the value of *Θ*_CW_ is found to be a larger than that of *T*_C_ which corresponds to the short-range order which may originate from the excess ion. The experimental effective magnetic moment has been calculated by using *C* values according to the following expression:^1^8
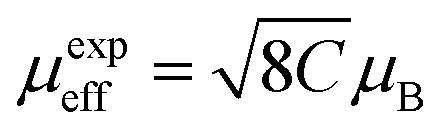


The calculated values of *μ*^exp^_eff_ are tabulated in Table 1 where the decreasing trend of *μ*^exp^_eff_ with an increase of Cr content has been observed. The decrease in *μ*^exp^_eff_ with the increase of Cr^3+^ content may refer to the decrease in ferromagnetic clusters present in the paramagnetic phase.^2^

The microwave frequency (*ω*_m_) is an important parameter for any materials for high-frequency microwave applications. The *ω*_m_ can be evaluated by using the following expression:^1,2^

9 *ω*_m_ = *γ*_1_8π^2^*M*_s_

where *γ*_1_ is the gyromagnetic ratio (*γ*_1_ = 2.8 MHz Oe^−1^). The calculated *ω*_m_ values are tabulated in Table 1, where maximum value is observed for *x* = 0.125. The calculated *ω*_m_ values are in the range of 8.1–15.6 GHz which is higher than that of previously reported values.^1,2^ Thus, it is affirmed that synthesized compositions may be a good candidate for high-frequency microwave applications such as satellite communications and biomedical applications.^44^

### 3.3 Critical scaling

#### 3.3.1 Modified Arrott plot

**Fig. 3:**
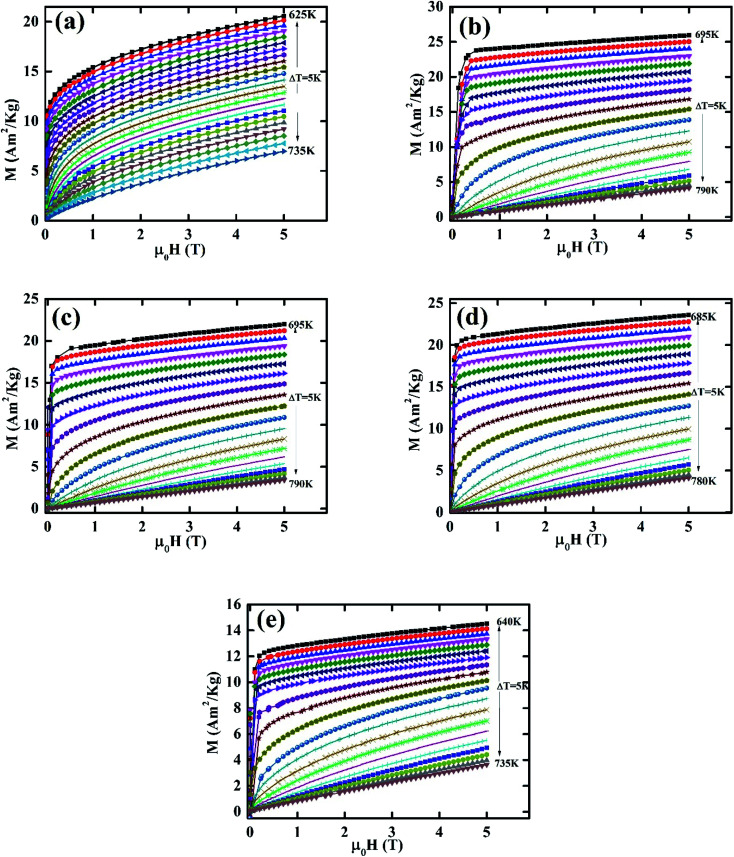
M–H isotherm for various Co_1−*x*_Cr_*x*_Fe_2_O_4_. (a) *x* = 0.0, (b) *x* = 0.125, (c) 0.25, (d) 0.375, and (e) *x* = 0.500.

The critical analysis is most important for any inorganic material close to the phase transition. The critical behavior has been conducted by measuring the magnetization isotherms (M–H) close to the respective *T*_C_ following the procedure described in ref. 4 and 34. The M–H isotherms for all samples are illustrated in Fig. 3. The non-linear M–H behavior below *T*_C_ confirms the compositions are ferromagnetic (FM), and the linear M–H behavior above *T*_C_ confirms paramagnetic (PM) nature. Based on the Landau theory, the Gibbs free energy can be written as.^45^10
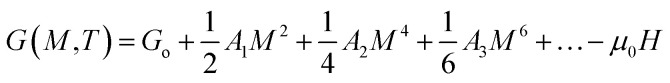
where, *A*_1_, *A*_2_, and *A*_3_ are Landau co-efficient. Neglecting the higher-order terms the above equation can be written as 11
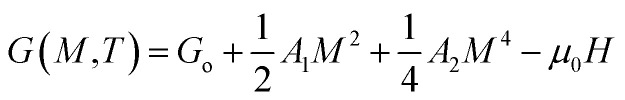


At the equilibrium condition, 
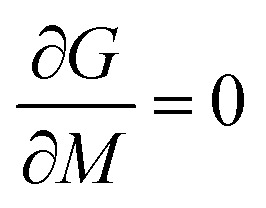
; then the magnetic equation of state can be written as12
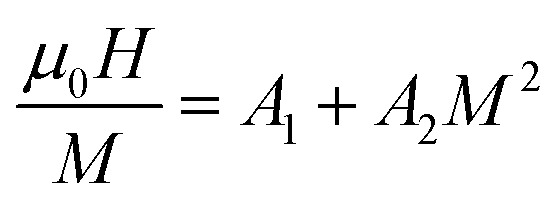


**Fig. 4:**
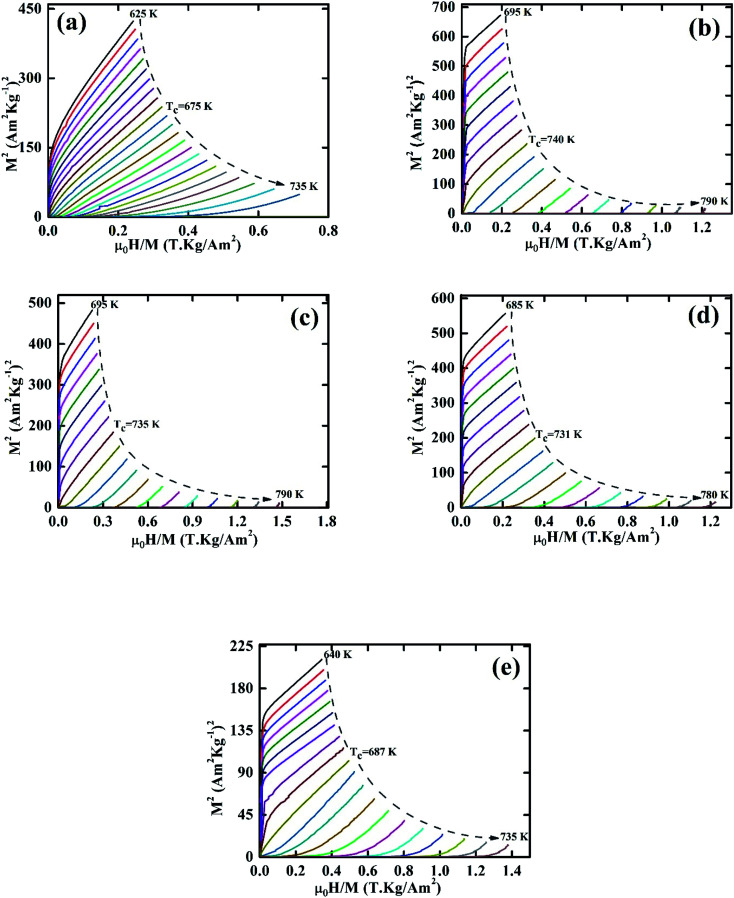
Arrott plots for various Co_1−*x*_Cr_*x*_Fe_2_O_4_. (a) *x* = 0.0, (b) *x* = 0.125, (c) 0.25, (d) 0.375, and (e) *x* = 0.500.

The nature of FM–PM phase transition may be determine from the *M*^2^*vs. μ*_0_*H*/*M*, known as Arrott plot.^36^ The Arrott plots for all samples are depicted in Fig. 4. No negative slope has been found in Fig. 4, which confirms the second-order phase transition. It is worth noting that *M*^2^*versus μ*_0_*H*/*M* plot should follow the equation of straight line passes through the origin. However, the above-mentioned behavior is not observed for all samples. Therefore, further analysis is performed for assumed second-order FM-PM phase transition using modified Arrott plots (MAP) according to Arrott–Noakes^37^ as mentioned by the following expression:13
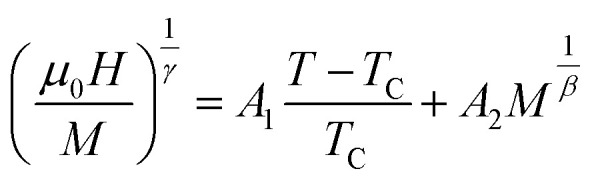
where *β*, and *γ* are the critical exponents.

The set of critical exponents (*β*, *γ*, and *δ*) are calculated by analyzing spontaneous magnetization (*M*_SP_), zero-field susceptibility (*χ*_0_), and magnetization isotherm at the *T*_C_ using the following power-laws:^4,34^

14 *M*_SP_(*T*) = *M*_0_(−*ε*)^*β*^, for *ε* < 0, *T* < *T*_C_,

15 *χ*_0_(*T*) = *Γ*(*ε*)^*γ*^, for *ε* > 0, *T* > *T*_C_

16 *M* = *D*_1_(*μ*_0_*H*)^1/*δ*^, for *ε* = 0, *T* = *T*_C_

17 *M*(*μ*_0_*H*,*ε*)|*ε*|^−*β*^ = *f*_±_*μ*_0_*H*|*ε*|^−(*β*+*γ*)^

where, 
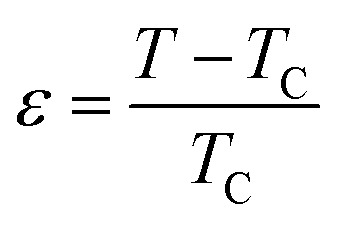
 is the reduced temperature, *M*_0_, *Γ*, and *D*_1_ are the critical coefficients, and *f*_+_ and *f*_−_ are the scaling functions above and below *T*_C_, respectively.

**Fig. 5:**
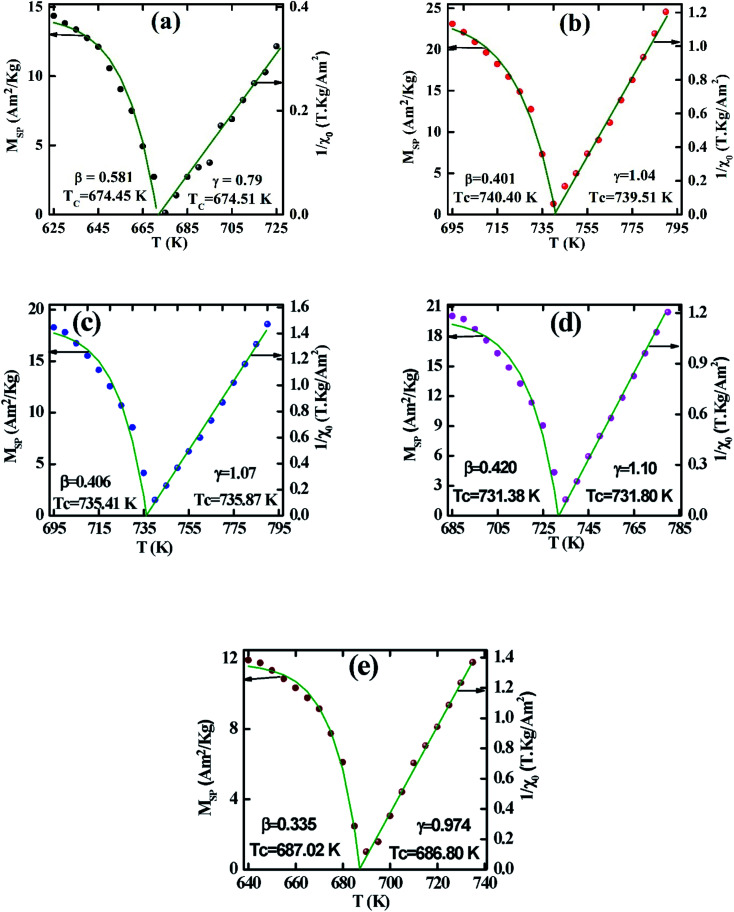
Spontaneous magnetization *M*_SP_ and zero field inverse susceptibility *χ*_0_^−1^ as a function of temperature for various Co_1−*x*_Cr_*x*_Fe_2_O_4_. (a) *x* = 0.0, (b) *x* = 0.125, (c) 0.25, (d) 0.375, and (e) *x* = 0.500.

**Fig. 6:**
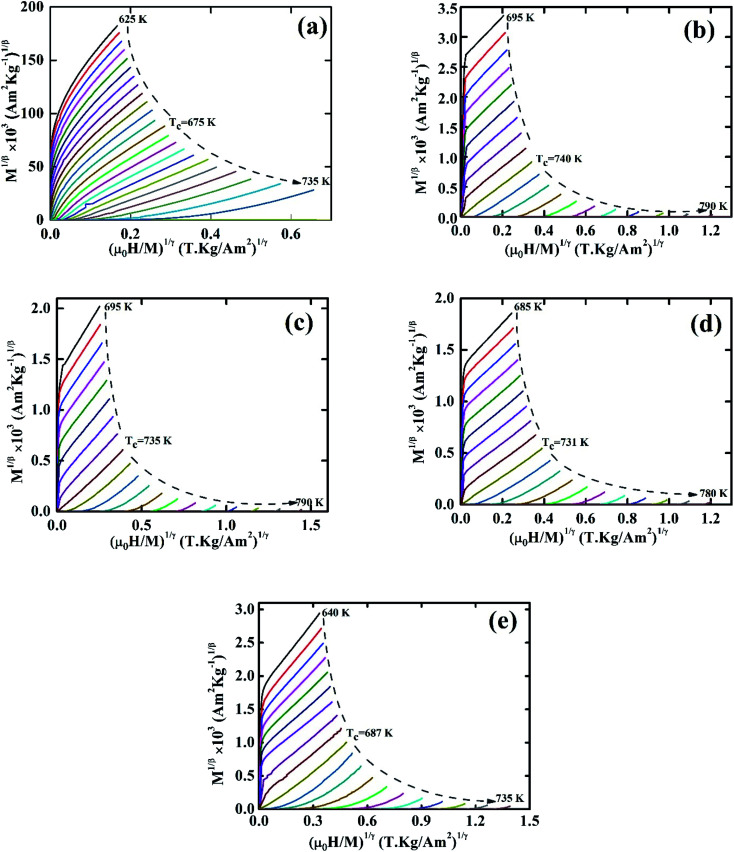
Modified Arrott plot for various Co_1−*x*_Cr_*x*_Fe_2_O_4_. (a) *x* = 0.0, (b) *x* = 0.125, (c) 0.25, (d) 0.375, and (e) *x* = 0.500.

To calculate the values of *β* and *γ* (using eqn (14) and (15)) the *M*_SP_*vs. T*, and 1/*χ*_0_*vs. T* are presented in Fig. 5. From Fig. 5 the *β* and *γ* values are estimated from the fitting curve for all the samples that have been tabulated in Table 2. From Table 2 it is found that the values of *β* and *γ* are close to the values of the mean-field model for the samples *x* = 0.125, 0.250, 0.375, however, for *x* = 0 and *x* = 0.5, there is a large difference that affects the MCE values as explained in the Section 3.4. The *T*_C_ values are also calculated from the fitting curve of Fig. 5 which also has been tabulated in Table 2 and the values are close to that obtained from M–T measurements described in Section 3.2. The 
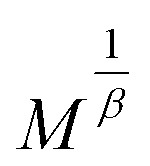
*vs.*
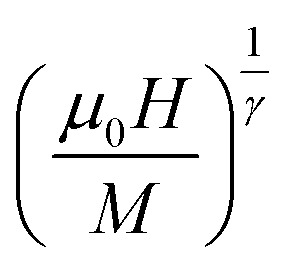
 graphs (MAP) for all samples are presented in Fig. 6 using the *β*, and *γ* values extracted from Fig. 5. These MAP plots show the straight line that passes through the origin at *T*_C_ for *x* = 0.125, 0.25, 0.375, and 0.500 which satisfy the required condition discussed earlier, however, *x* = 0 shows dissimilar behavior.

**Table 2:** The obtained values of critical exponents (*β*, *γ*, and *δ*) and *T*_C_s from the modified Arrott plot (MAP), Kouvel–Fisher (KF) plot, critical isotherm, Widom scaling, magnetocaloric effect, and relative cooling power (RCP) analysis across the PM–FM transition region for various Co_1−*x*_Cr_*x*_Fe_2_O_4_

*x*	*β*	*γ*	*δ*	*n*	*T*_C_ (K)	Methods
0.000	0.581	0.79	—	—	674.45	MAP
					674.51	
	0.325	1.17	—	0.231	673.37	KF
					673.42	
	—	—	3.23	—	—	Critical isotherm
	—	—	2.34	—	—	MCE/RCP
	—	—	—	0.910	675	|Δ*S*^max^_m_|
	—	—	2.36	—	—	Widom scaling
0.125	0.401	1.04	—	—	740.4	MAP
					739.5	
	0.461	0.998	—	0.676	740.12	KF
					740.09	
	—	—	3.61	—	—	Critical isotherm
	—	—	3.64	—	—	MCE/RCP
	—	—	—	0.677	740	|Δ*S*^max^_m_|
	—	—	3.59	—	—	Widom scaling
0.250	0.406	1.07	—	—	735.41	MAP
					735.87	
	0.464	1.03	—	0.685	734.92	KF
					735.09	
	—	—	3.62	—	—	Critical isotherm
	—	—	3.65	—	—	MCE/RCP
	—	—	—	0.690	735	|Δ*S*^max^_m_|
	—	—	3.63	—	—	Widom scaling
0.375	0.42	1.1	—	—	731.38	MAP
					731.80	
	0.466	1.01	—	0.682	731.32	KF
					730.89	
	—	—	3.60	—	—	Critical isotherm
	—	—	3.61	—	—	MCE/RCP
	—	—	—	0.679	731	|Δ*S*^max^_m_|
	—	—	3.62		—	Widom scaling
0.500	0.335	0.974	—	—	687.02	MAP
					686.80	
	0.331	1.08	—	0.422	686.85	KF
					686.89	
	—	—	3.50	—	—	Critical isotherm
	—	—	4.43	—	—	MCE/RCP
	—	—	—	0.501	687	|Δ*S*^max^_m_|
	—	—	3.91	—	—	Widom scaling

**Fig. 7:**
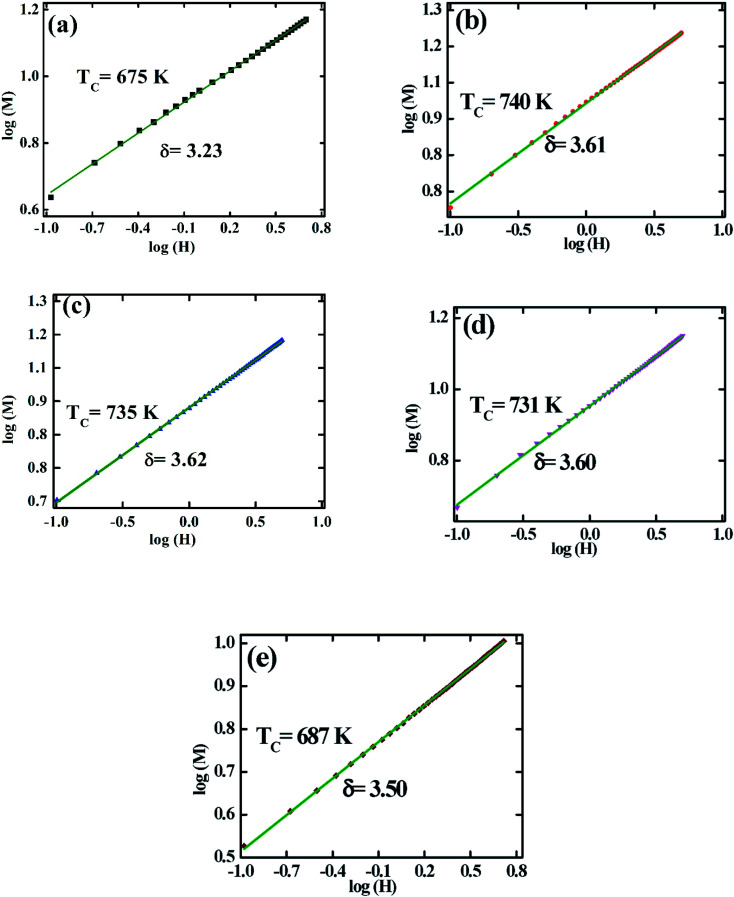
The log–log plot of isothermal magnetization (*M*) *vs.* applied field (*H*) for various Co_1−*x*_Cr_*x*_Fe_2_O_4_. (a) *x* = 0.0, (b) *x* = 0.125, (c) 0.25, (d) 0.375, and (e) *x* = 0.500.

The variation in the critical isotherm *M*(*T*_C_,*H*) can be described by a power-law (eqn (16)) characterized by the critical exponent *δ*. The critical exponent *δ* has been obtained from the inverse of slopes of *M*(*T*_C_) *vs. H* curve in the log–log scale as shown in Fig. 7. The *δ* values are also determined from the previously calculated *β*, and *γ* values according to statistical theory using Widom relation:^46^18
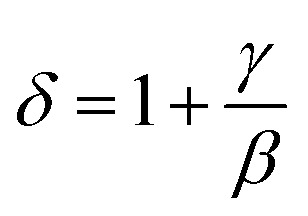


The estimated *δ* values according to the above two cases for all samples are tabulated in Table 2. The *δ* values for both cases are close to each other for the sample for *x* = 0.125, 0.25, and 0.375 universal class. But for *x* = 0.00, and 0.500 the *δ* values do not match each other.

#### 3.3.2 Kouvel–Fisher plot

**Fig. 8:**
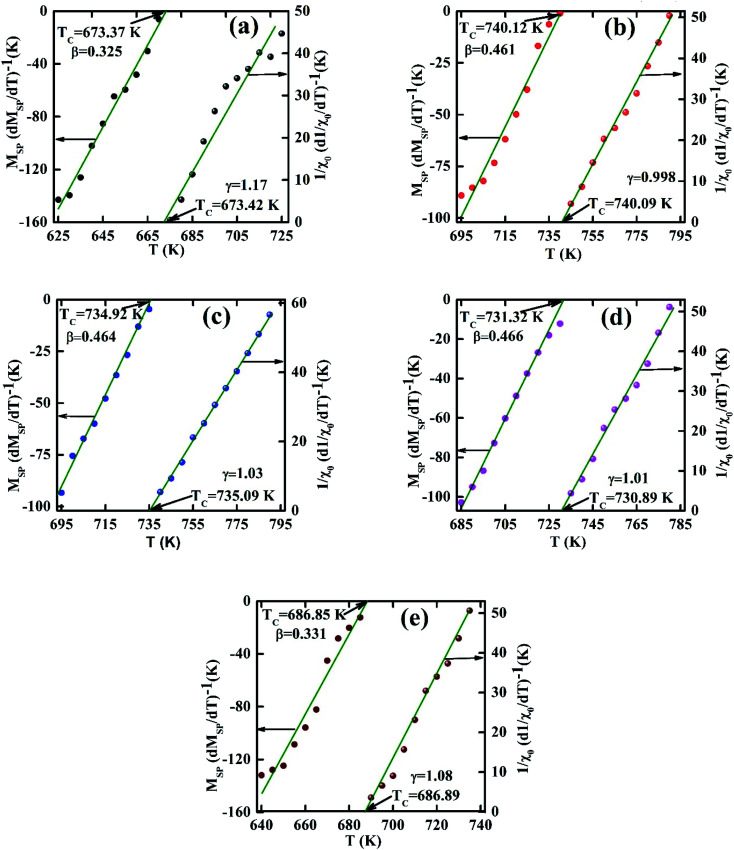
Kouvel–Fisher plots *M*_SP_ and *χ*_0_^−1^ as a function of temperature for various Co_1−*x*_Cr_*x*_Fe_2_O_4_. (a) *x* = 0.0, (b) *x* = 0.125, (c) 0.25, (d) 0.375, and (e) *x* = 0.500.

The *β*, *γ*, and *T*_C_ have been calculated from the Kouvel–Fisher plots (KFPs) that provide more reliable values.^47^ In KFPs *β*, *γ*, and *T*_C_ has been extracted from 
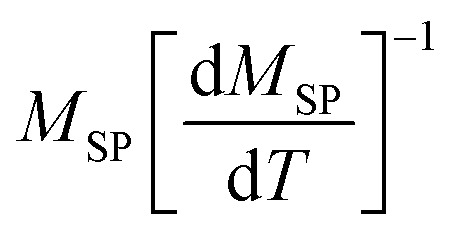
 and 
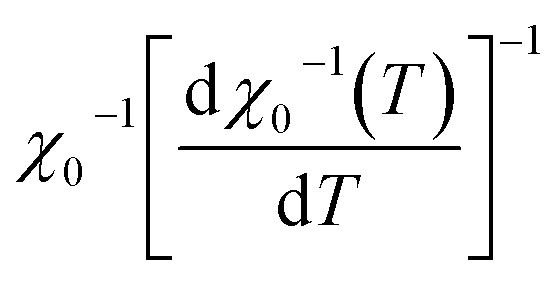
*vs. T* graph (Fig. 8) according to the following expressions:^34^19
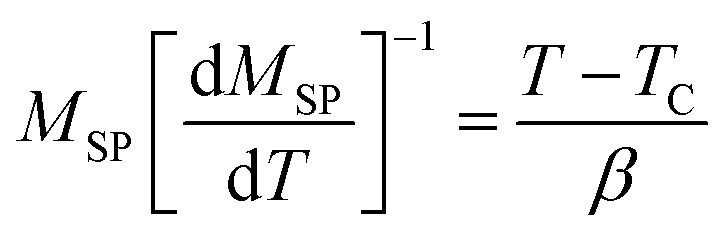
20
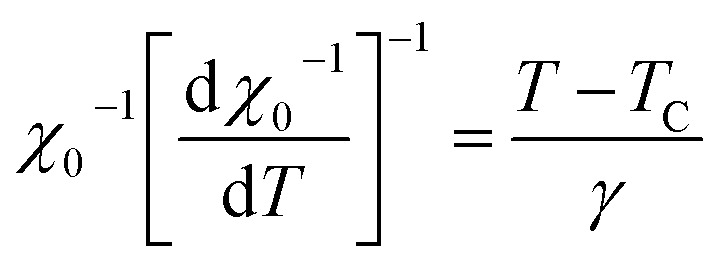


*T*_C_ values are extracted from the X-intercepts, and critical *β* and *γ* values are obtained from the inverse of slopes of the fitted straight line of Fig. 8. The estimated *β*, *γ*, and *T*_C_ values for all the samples according to KFPs are tabulated in Table 2, where *β*, *γ*, and *T*_C_ values are well-matched with the values as the mean-field theory for *x* = 0.125, 0.250, and 0.375. However, for *x* = 0.00, and 0.500 the *β*, and *γ* values calculated from KFPs show a remarkable difference compared to that of mean-field theory.

**Fig. 9:**
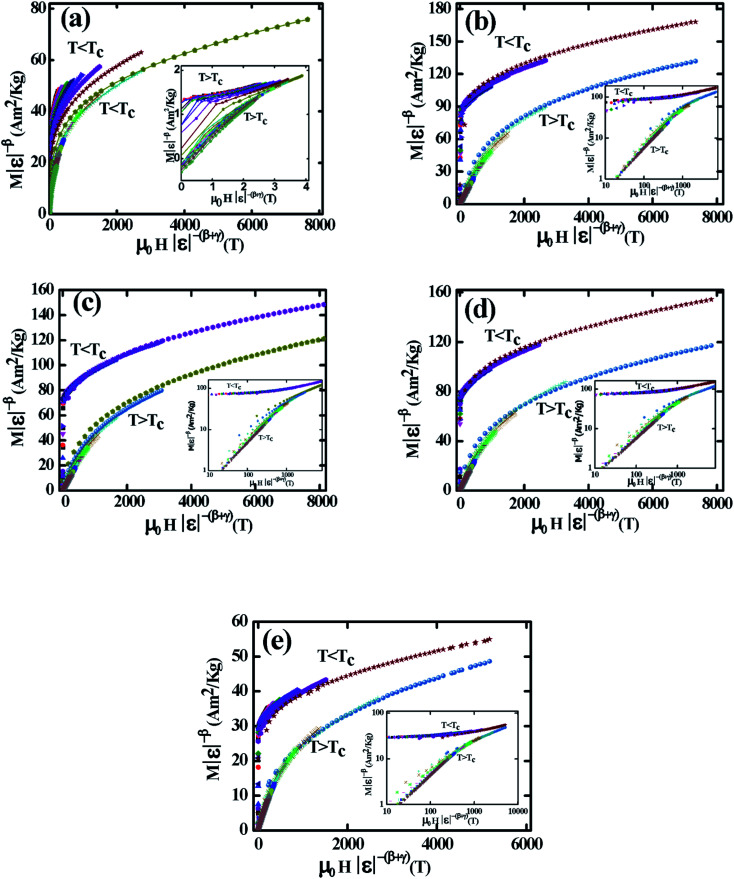
Scaling plots below and above *T*_C_ values using *β* and *γ* estimated using the Kouvel–Fisher method. Insets show plots in the log–log scale for various Co_1−*x*_Cr_*x*_Fe_2_O_4_. (a) *x* = 0.0, (b) *x* = 0.125, (c) 0.25, (d) 0.375, and (e) *x* = 0.500.

To ensure the reliability of *β*, *γ*, and *T*_C_ values another robust method have been elucidated by plotting *M*|*ε*|^−*β*^*vs. μ*_0_*H*|*ε*|^−(*β*+*γ*)^) just above and below *T*_C_ according to eqn (17). The *M*|*ε*|^−*β*^*vs. μ*_0_*H*|*ε*|^−(*β*+*γ*)^) have been plotted for all samples in Fig. 9. The inset in Fig. 9, each case displays the same data plotted on a log–log scale. From Fig. 9, it is evident that two separate groups of isotherms superimpose (one group greater than *T*_C_, and the other group less than *T*_C_) for the samples *x* = 0.125, 0.250, and 0.375. These results suggest the accuracy of *β*, *γ*, and *T*_C_ values from which it can be decided that these three compositions (*x* = 0.125, 0.250, and 0.375) are a universal class of material. From the inset of Fig. 9, two branches (one below *T*_C_ and other above *T*_C_) show the linear behavior in the high field region while in the low field region show some deviation from linearity. These behaviors confirm that the scale theory gives more important data in higher fields. The isotherms for *x* = 0.00, and 0.500 show different behavior that imply the non-universal class of the materials.

### 3.4 Magnetocaloric effect

The MCE properties is an intrinsic properties of magnetic materials that can be calculated by calculating the magnetic entropy change (Δ*S*_m_) around *T*_C_. The Δ*S*_m_ values are calculated from the isothermal M–H data based on Maxwell's thermodynamic relation:^34^21
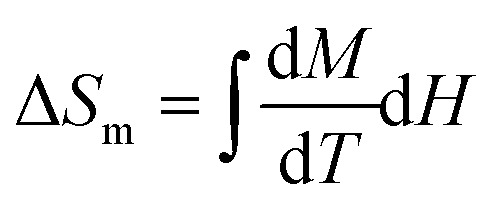


**Fig. 10:**
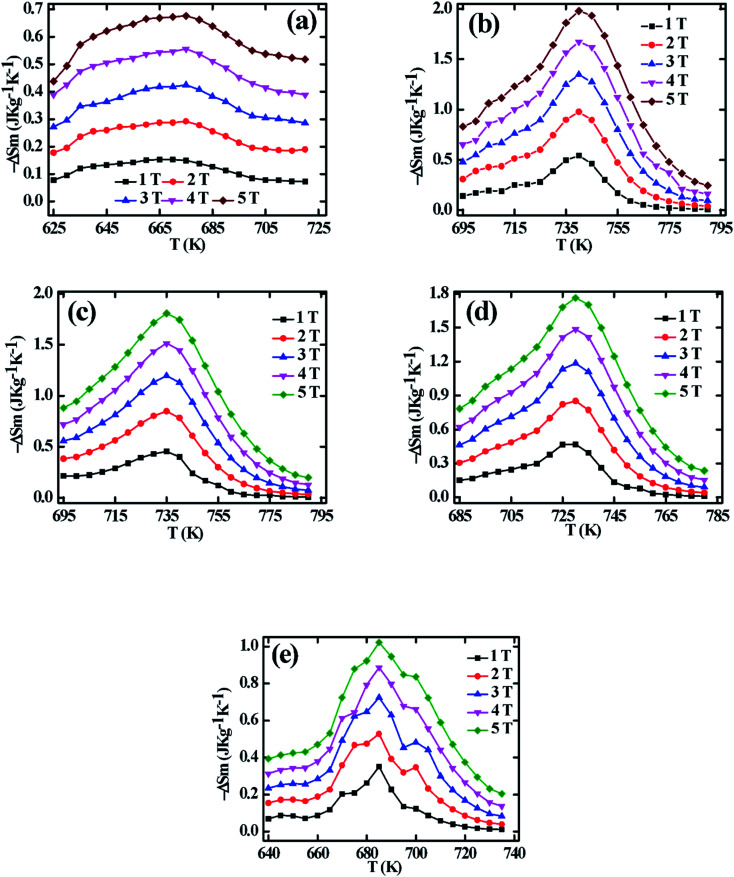
Magnetic entropy change as a function of temperature for various Co_1−*x*_Cr_*x*_Fe_2_O_4_. (a) *x* = 0.0, (b) *x* = 0.125, (c) 0.25, (d) 0.375, and (e) *x* = 0.500.

The calculated Δ*S*_m_ for all samples show negative values for all temperature and applied magnetic field. The calculated −Δ*S*_m_ values as a function of temperature are illustrated in Fig. 10 for all samples at different magnetic fields up to 5 T. From Fig. 10, the peak values of −Δ*S*_m_ are defined as maximum entropy change |Δ*S*^max^_m_| are evident at *T*_C_ or close to *T*_C_. From Fig. 10 it is observed that |Δ*S*^max^_m_| increases with an increase of magnetic field are due to the spin ordering for all the samples. The |Δ*S*^max^_m_| values are tabulated in Table 3 for 5 T for all samples. Table 3 shows that for *x* = 0.125, maximum entropy change is observed, however, a decreasing trend is found for further increasing of Cr content. The similar behavior is observed for *M*_s_.

**Table 3:** Comparison of the Curie temperature, |Δ*S*^max^_m_| and RCP for various Co_1−*x*_Cr_*x*_Fe_2_O_4_ and some other reported samples

Samples	*T*_C_ (K)	*μ*_0_ *H* (T)	|Δ*S*^max^_m_| J kg^−1^ K^−1^	RCP (J kg^−1^)	Reference
CoFe_2_O_4_	675	5	0.66	35.7	This work
Co_0.875_Cr_0.125_Fe_2_O_4_	740	5	1.98	128	
Co_0.75_Cr_0.25_Fe_2_O_4_	735	5	1.8	137	
Co_0.625_Cr_0.375_Fe_2_O_4_	731	5	1.76	145	
Co_0.5_Cr_0.5_Fe_2_O_4_	687	5	1.02	52	
Ni_0.6_Cd_0.2_Cu_0.2_Fe_2_O_4_	680	5	2.12	125	Ref. 4
Zn_0.4_Ni_0.2_Cu_0.4_Fe_2_O_4_	565	5	1.41	141	Ref. 29
Ni_0.4_Mg_0.3_Cu_0.3_Fe_2_O_4_	690	5	1.56	136	Ref. 30
Zn_0.25_Ni_0.25_Mg_0.5_Fe_2_O_4_	590	5	1.16	90	Ref. 31
Mg_0.6_Cu_0.2_Ni_0.2_Fe_2_O_4_	670	5	1.38	137	Ref. 32
Mg_0.6_Cu_0.4_Fe_2_O_4_	630	5	1.09	136	Ref. 32

Relative Cooling Power (RCP) is another important criterion that helps to characterize the MCE of such magnetic materials. The RCP for all samples has been calculated using the following relation:^4^

22 RCP = |Δ*S*^max^_m_| × *δT*_FWHM_

where *δT*_FWHM_ is the full width of the 0.5|Δ*S*^max^_m_|. The calculated RCP values are tabulated in Table 3, where very lower values of RCP with lower |Δ*S*^max^_m_| for *x* = 0.00 are evident which may be due to the non-universal nature as explained in Section 3.3. For *x* = 0.125, 0.250, and 0.375 it show the comparable values of RCP reported earlier for various ferrite materials.^4,29–32^ From Table 3, RCP values are found to increase with the increasing Cr content and found a maximum of 145 J kg^−1^ for *x* = 0.375 which is higher than the previously reported RCP values.^4,29–32^ For *x* = 0.500 the RCP values are found to be very low which may be due to showing negative values of the vacancy parameter as explained in the previously reported article.^25^ Another reason behind showing the lower values of RCP is non-universal behavior for *x* = 0.500.

To analyze the critical exponent, the magnetic field dependent |Δ*S*^max^_m_| and RCP are fitted according to the following power law:^38^

23 |Δ*S*^max^_m_| ∝ (*μ*_0_*H*)^*n*^

**Fig. 11:**
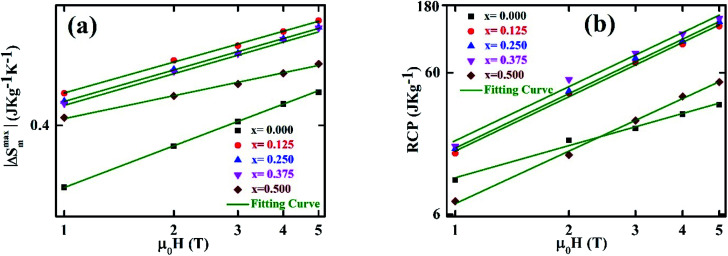
Magnetic field dependence (a) |Δ*S*^max^_m_|at *T*_C_ fitted to the power law |Δ*S*^max^_m_| ∝ (*μ*_0_*H*)^*n*^, (b) RCP at *T*_C_ fitted to the power law RCP ∝ (*μ*_0_*H*)^1+1/*δ*^.

24
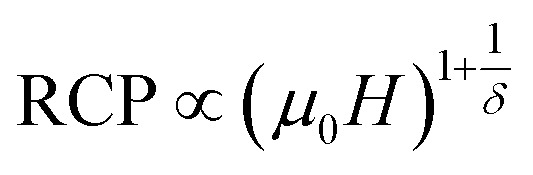
where, *n* is the exponent that depends on the magnetic state of the samples. The |Δ*S*^max^_m_| *vs. μ*_0_*H* are plotted in the log–log scale and illustrated in Fig. 11(a) for all samples, and the values of *n* are obtained from the slope of the linear fitting. The obtained *n* values have been tabulated in Table 2. To explain the reliability of this exponent, calculated the value of *n* at/near *T*_C_ by using the following relation:^34^25
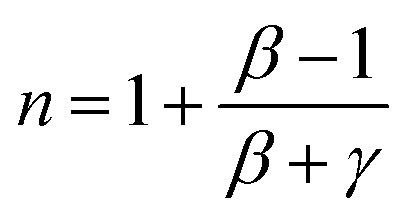


By applying the Widom relation eqn (18) and (25) can be rewritten as26
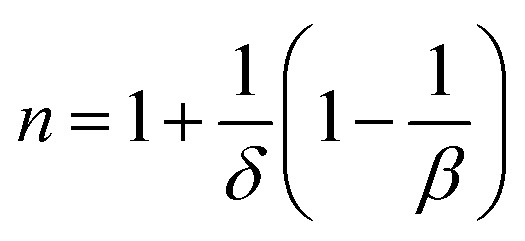


The calculated *n* exponents (using eqn (26)) have been tabulated in Table 2 for all compositions. In this case *β* and *γ* values are considered from KFPs, and values of *δ* are considered from the critical isotherms. The exponent calculated from eqn (26) are in good agreement with those obtained from the fitted curve of Fig. 11(a) for *x* = 0.125, 0.250, and 0.375. For *x* = 0.00, and 0.500, there is a large difference in the value of *n*.

The *δ* values have been obtained from the slope of the linear fit of the RCP *vs. μ*_0_*H* plot in the log–log scale (Fig. 11(b)) according to eqn (24). The obtained *δ* values by this method are tabulated in Table 2, from where *δ* values show a good agreement with those of *δ* values obtained from Widom scaling and critical isotherms for the samples *x* = 0.125, 0.25, and 0.375 (Sec. 3.3). However, for *x* = 0.00, 0.500 a large difference of *δ* values has been obtained. Comparing the values of *n* and *δ* according to Fig. 11, and critical scaling it is decided that the MCE properties of present compositions are reliable.

Verification of critical phenomena and the nature of the magnetic phase transition of these materials are also important. In 2006, Franco *et al.* have utilized the phenomenological universal scaling curve.^39^ According to this method normalized magnetic entropy as a function of re-scaled temperature *θ* (eqn (27)) has been plotted at several magnetic fields which are depicted in Fig. 12.27
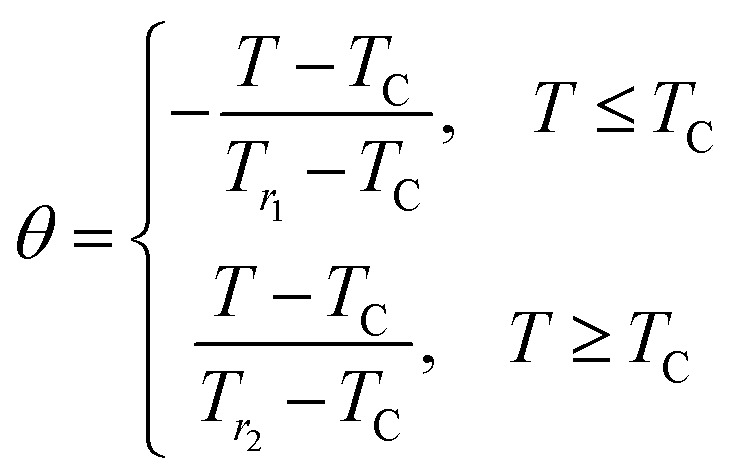
where, *T*_r_1__ and *T*_r_2__ are two-temperatures corresponding to 0.5|Δ*S*^max^_m_|.

**Fig. 12:**
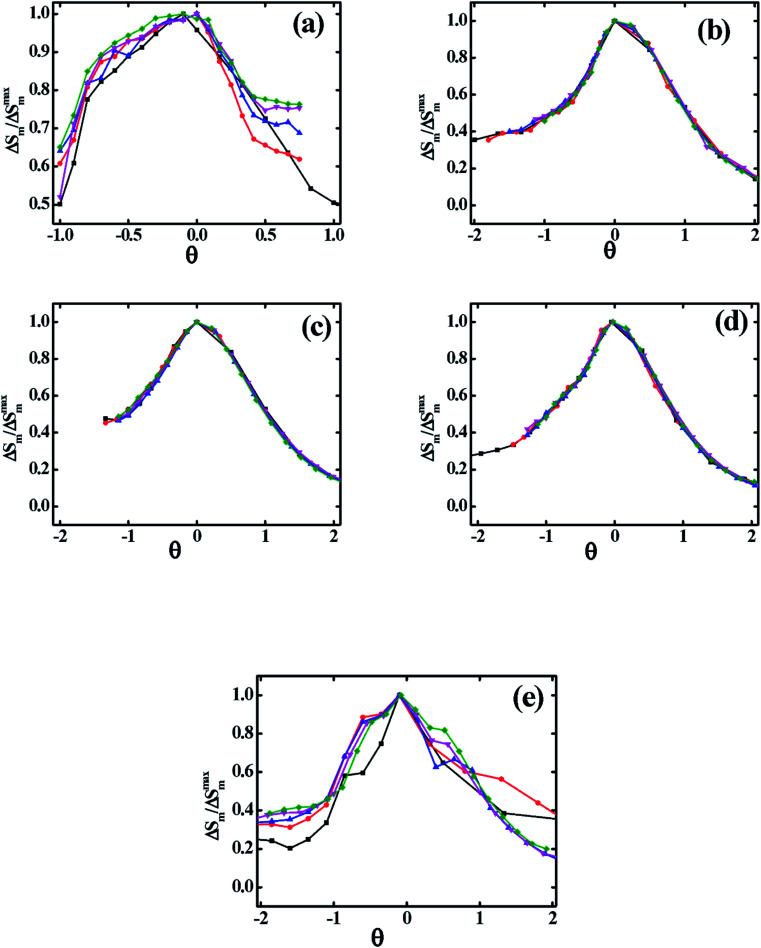
Normalized magnetic entropy change as function of rescaled temperature for various Co_1−*x*_Cr_*x*_Fe_2_O_4_. (a) *x* = 0.0, (b) *x* = 0.125, (c) 0.25, (d) 0.375, and (e) *x* = 0.500.

In Fig. 12(b–d) it is observed that the results of various magnetic field collapsed into a single master curve, which implies that synthesized samples of *x* = 0.125, 0.250, and 0.375 are in universal class and show exact second-order phase transition.^34^ However, from Fig. 12(a and e) it is evident that for *x* = 0.00, and 0.500, samples are non-universal class.

To analyze the accuracy of MCE properties and order of phase transition, the value of *n* is calculated using the following expression:^38^28
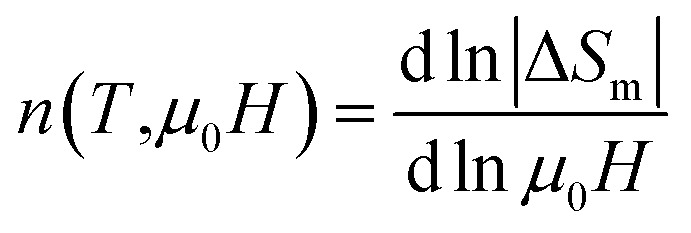


**Fig. 13:**
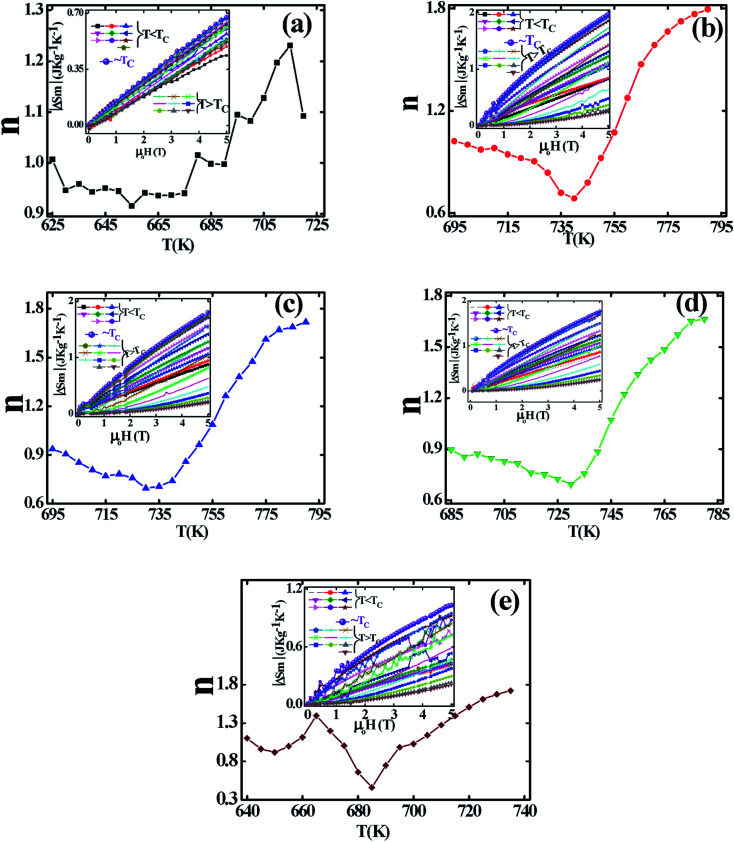
The exponent n as function of temperature obtained from the fitting of field dependent isothermal magnetic entropy change at various temperature for various Co_1−*x*_Cr_*x*_Fe_2_O_4_. (a) *x* = 0.0, (b) *x* = 0.125, (c) 0.25, (d) 0.375, and (e) *x* = 0.500.

The calculated *n* values as a function of *T* are illustrated in Fig. 13 for all samples where the inset depicted the |Δ*S*_*m*_| *vs. μ*_0_*H*. From the Fig. 13(b–d), it is found that the *n* values for *x* = 0.125, 0.250, 0.375 are close to 1 below *T*_C_ which suggests that the d*M*/d*T* term in eqn (21) is weakly field-dependent.^34^ With an increase in temperature it is observed the decreasing trend and arrive the minimum *n* values of 0.684, 0.695, and 0.693 at *T*_C_ for *x* = 0.125, 0.25, and 0.375, respectively. These *n* values are consistent with the *n* values obtained from Fig. 11(a) and also from eqn (26) as tabulated in Table 2. Above *T*_C_, the *n* values are found to be the increasing trend but do not cross the critical value of 2 for *x* = 0.125, 0.250, and 0.375. The minimum *n* values at *T*_C_ and *n* < 2 above *T*_C_ confirm the second-order phase transition which is explained by Law *et al.*^38^ For *x* = 0.00, and 0.500 as evident from Fig. 13(a and e) it is found the anomalous behavior of *n*-T curves shows the minimum *n* values at *T*_C_ which is very different compared with that of the *n* values described in Sec. 3.3. This behavior for *x* = 0.00, and 0.500 is non-universal class of materials showing the non-realistic MCE values. Although *n*-T shows anomalous behavior, however, *n* values less than 2 for all the temperature suggest that the samples (*x* = 0.00, and 0.500) exhibit the second-order phase transition.

## 4 Conclusions

The effect of Cr^3+^ substitution on the magnetic and MCE properties of various Co_1−*x*_Cr_*x*_Fe_2_O_4_ prepared by the solid–state reaction technique have been evident in this report. The Arrott plot from the analysis of M–H isotherms exhibits the second-order phase transition that has been perfectly confirmed from the critical analysis and scaling analysis of the MCE effect. The *x* = 0.125, 0.250, and 0.375 samples demonstrate high RCP values in the range of 127–145 J kg^−1^ compared to that of other ferrites. The universal curve scaling and scaling analysis of the MCE effect confirms the universal class and the MCE values for *x* = 0.125, 0.250, and 0.375 are reliable. The higher MCE values up to 145 J kg^−1^ are observed for *x* = 0.375, which might be considered as potential candidates for the cooling technology. On the other hand, the higher microwave frequency for all compositions makes them a strong candidate for high-frequency microwave applications, especially in satellite communications and biomedical applications.

## Conflicts of interest

There are no conflicts to declare.

## Supplementary Material
